# Epidemiological characteristics and prediction model construction of hemorrhagic fever with renal syndrome in Quzhou City, China, 2005–2022

**DOI:** 10.3389/fpubh.2023.1333178

**Published:** 2024-01-11

**Authors:** Qing Gao, Shuangqing Wang, Qi Wang, Guoping Cao, Chunfu Fang, Bingdong Zhan

**Affiliations:** ^1^School of Public Health, Zhejiang Chinese Medical University, Hangzhou, Zhejiang, China; ^2^Quzhou Center for Disease Control and Prevention, Quzhou, Zhejiang, China

**Keywords:** hemorrhagic fever with renal syndrome, spatiotemporal analysis, land use, SARIMA, Prophet

## Abstract

**Background:**

Hemorrhagic fever with renal syndrome (HFRS) is one of the 10 major infectious diseases that jeopardize human health and is distributed in more than 30 countries around the world. China is the country with the highest number of reported HFRS cases worldwide, accounting for 90% of global cases. The incidence level of HFRS in Quzhou is at the forefront of Zhejiang Province, and there is no specific treatment for it yet. Therefore, it is crucial to grasp the epidemiological characteristics of HFRS in Quzhou and establish a prediction model for HFRS to lay the foundation for early warning of HFRS.

**Methods:**

Descriptive epidemiological methods were used to analyze the epidemic characteristics of HFRS, the incidence map was drawn by ArcGIS software, the Seasonal AutoRegressive Integrated Moving Average (SARIMA) and Prophet model were established by R software. Then, root mean square error (RMSE) and mean absolute error (MAE) were used to evaluate the fitting and prediction performances of the model.

**Results:**

A total of 843 HFRS cases were reported in Quzhou City from 2005 to 2022, with the highest annual incidence rate in 2007 (3.93/100,000) and the lowest in 2022 (1.05/100,000) (*P* trend<0.001). The incidence is distributed in a seasonal double-peak distribution, with the first peak from October to January and the second peak from May to July. The incidence rate in males (2.87/100,000) was significantly higher than in females (1.32/100,000). Farmers had the highest number of cases, accounting for 79.95% of the total number of cases. The incidence is high in the northwest of Quzhou City, with cases concentrated on cultivated land and artificial land. The RMSE and MAE values of the Prophet model are smaller than those of the SARIMA (1,0,1) (2,1,0)12 model.

**Conclusion:**

From 2005 to 2022, the incidence of HFRS in Quzhou City showed an overall downward trend, but the epidemic in high-incidence areas was still serious. In the future, the dynamics of HFRS outbreaks and host animal surveillance should be continuously strengthened in combination with the Prophet model. During the peak season, HFRS vaccination and health education are promoted with farmers as the key groups.

## 1 Introduction

Hantavirus (HV) is a single-stranded enveloped RNA virus in the Bunyaviridae family. It primarily causes two infectious diseases in humans, namely Hantavirus Cardiopulmonary Syndrome (HCPS) in the Americas and hemorrhagic fever with renal syndrome (HFRS) in Europe and Asia (1). The morbidity and mortality rates vary from 0 to 43% among various pathogenic virus genotypes (2). Global HV infections show a fluctuating upward trend (3). HFRS is a naturally zoonotic disease, with rodents and other small mammals as the main source of transmission and natural hosts, the *Apodemus agrarius* and *Rattus norvegicus*, which carry HVs known as hantaan virus (HTVN) and Seoul virus (SEOV), respectively. The virus is mainly transmitted by aerosol or direct contact (4). The main clinical manifestations include kidney damage, bleeding, and fever (5). At present, HFRS occurs in more than 30 countries in the world, such as China, the United States, Russia, etc. (6–8). It can report 150,000–200,000 cases annually and is a global public health problem (9). According to statistics, China has the largest number of HFRS cases in the world, with an annual report of 20,000–50,000 cases, accounting for 90% of the global cases (10, 11). It poses a serious threat to the health of residents and socio-economic development and has become one of the major public health problems (12).

HFRS cases were first detected in Zhejiang Province in 1963, and outbreaks occurred in many areas of southern China in the 1980s. Zhejiang Province has a high incidence of HFRS in southern provinces, China. In the past decade, outbreaks in Zhejiang Province have continued to be sporadic. Quzhou, located in the western part of Zhejiang Province, has always been a high-incidence area, and Kaihua County ranks among the top five in the province in terms of cumulative cases from 2005 to 2020 (13). Therefore, it is of great significance to establish an epidemic prediction model for HFRS, explore its epidemic characteristics and development patterns, and seek optimal prevention and control strategies.

The prevalence of HFRS has been found to be influenced by various aspects, such as the accelerated progress of urbanization, and changes in land use types may also increase the risk of HFRS (14). Different populations have different probabilities of contact with rodents, leading to different risks of infection (15). Meanwhile research evidence suggests that a significant decline in the incidence of vector-borne infections occurred as a result of the 2020 policy of non-pharmacological interventions for novel coronavirus pneumonia. And there is no specific treatment for HFRS (16). In recent years, many scholars have used time series analyses to study the temporal changes of HFRS in order to predict future trends, such as the product seasonal autoregressive moving average model (SARIMA) (17). The model has a simple structure, requires only morbidity information for prediction, is highly feasible, and has been widely used in healthcare (18). The Prophet model is a procedure for predicting time series data based on an additive model, which can simulate multiple seasons simultaneously by means of a generalized additive model, and the fitting effect has been validated in time series prediction of infectious diseases such as Hand, Foot and Mouth Disease (HFMD) and AIDS (19, 20), but the effect of the fit in HFRS is not yet conclusive.

In order to better understand the epidemiological characteristics of HFRS in Quzhou, improve the prevention and control measures, and optimize the allocation of resources. We conducted a descriptive analysis on the incident data of HFRS from 2005 to 2022. Using GIS technology, combining with land use data, we analyzed the spatial distribution characteristics of HFRS in Quzhou. The SARIMA and Prophet models were used to establish a prediction model for the reported incidence of HFRS in Quzhou City from 2005 to 2022, to compare the prediction effects of each model, to explore the optimal model, and to provide scientific suggestions for prediction and early warning in the prevention and control of HFRS in Quzhou City.

## 2 Methods

### 2.1 Setting

Quzhou is located in the western region of Zhejiang Province, China, the upper reaches of the Qiantang River, between 118°01′15″–119°20′20″ east longitude and 28°15′26″–29°30′00″ north latitude. The city’s terrain is dominated by mountainous hills, which is a subtropical monsoon climate, with four distinct seasons throughout the year, abundant sunshine and precipitation. By 2023, the city has jurisdiction over 2 districts, 3 counties, and 1 county-level city.

### 2.2 Data sources and collection

Data of confirmed HFRS cases in Quzhou from January 2005 to December 2022 were obtained from the National Infectious Disease Reporting System (NIDRS) and the Chinese Center for Disease Control and Prevention (CDC). Cases were reported by medical and health institutions at all levels in Quzhou and reviewed by CDC at all levels. All cases were confirmed according to the HFRS diagnostic criteria of the Ministry of Health of the People’s Republic of China (WS 278–2008). Confirmed cases must have a history of traveling to an infected area or a history of direct or indirect contact or suspected contact with rats or their excrement (stool, urine) components within 2 months prior to the onset of disease, and have at least two of the following clinical characteristics: fever, chills, bleeding, headache, back pain, abdominal pain, acute renal insufficiency, and hypotension. In addition, a laboratory diagnostic criterion must be met: a positive result for hantavirus-specific immunoglobulin M, a 4-fold rise in titers of hantavirus-specific immunoglobulin G, a positive result for hantavirus-specific ribonucleic acid by reverse transcription polymerase chain reaction in clinical specimens, or having hantavirus isolated from clinical specimens. Land use data from GlobeLand30 2020 edition,^1^ which was developed by the spatial resolution of 30 meters surface coverage data around the world, it divides land into 10 first-level types: arable land, Woodland, grassland, shrub land, wetland, water body, tundra, artificial surface, unused land, glacier and permanent snow cover. Population data were obtained from Quzhou Statistical Yearbook. The urban (county) boundary maps and township boundary maps of Quzhou are provided by the CDC.

### 2.3 Statistical analysis

#### 2.3.1 Descriptive analysis

Microsoft Excel (Microsoft Corporation, Redmond, WA, United States) was used to generate a database, organize and analyze the cases, and calculate the incidence rate. R software (version 4.2.1, R Foundation for Statistical Computing, Vienna, Austria) was used to analyze the prevalent characteristics of HFRS (time distribution, age distribution, sex distribution and occupation distribution) in Quzhou city from 2005 to 2022. Qualitative data were described as frequency and percent, and χ^2^-test was used for inter-group comparison, with *p* < 0.05 indicating a statistically significant difference.

#### 2.3.2 Spatiotemporal analysis

The incidence data of Quzhou City were divided by township/street, and the incidence rate was calculated. The annual average incidence map and cumulative incidence map from 2005 to 2022 were established on the township/street scale using GIS technology in ArcGIS software (version 10.8; ESRI Inc., Redlands, CA, United States), and the spatial distribution of HFRS in Quzhou City was dynamically displayed. The land use of Quzhou City was collected by GlobeLand30, and the incidence data were linked to draw the HFRS incidence maps of different land use types.

#### 2.3.3 Model construction

Autoregrestic moving average model (ARIMA) is a classical time series prediction method proposed by Geogre Box and Gwilym Jenkins, and has been widely used in the field of public health (21–23). ARIMA (p, d, q) model is a hybrid model consisting of auto regressive (AR) model and moving average (MA) model, p is the autoregressive order, q is the moving average order, and d is the difference order. On the basis of ARIMA model, SARIMA adds seasonal parameters, P is the order of seasonal autoregression, D is the order of seasonal difference, Q is the order of seasonal moving average, and S is the time length of seasonal cycle (24). The general form of the model is (p, d, q) × (P, D, Q) S (25), which is suitable for both trend and seasonality. The model construction is divided into four steps: 1. Stationarity test: The application of SARIMA model requires time series to meet the requirements of stationarity, and the stationarity of data is preliminarily observed according to the incidence time series diagram; Augmented Dickey-Fuller (ADF) test is carried out. If *p* > 0.05 and the sequence is not stable, the original sequence needs to be stabilized by difference and/or seasonal difference and variable transformation. 2. Model identification order: According to the characteristics of the auto correlation function (ACF) graph and partial auto correlation function (PACF) graph, the order is preliminatively determined and multiple models are compared. The optimal model is selected according to Akaike’s information criterion (AIC). 3. Model parameter estimation and diagnosis: parameter estimation and hypothesis test are performed on the identified model, and white noise (Ljung-box, LB) test is performed on the residual sequence, and *p* > 0.05 passes the test, indicating that it could be used for prediction. 4. Model fitting prediction.

Prophet model is a time series model developed by Facebook in 2017 based on the C++ programming language, which can be used in Python and R (26). The model can handle flexibly the missing values of data, as well as the effects of holidays and special events in time series. The fitting speed of the model is fast, and it is suitable for predicting time series with strong seasonal influence and historical data of multiple seasons (19). The general form of Prophet model is y(t) = g(t) + s(t) + h(t) + ε, g(t) is the trend function, s(t) is the periodic function, h(t) is the holiday function, ε is the error, with the addition model for accumulation. Model construction is mainly divided into four steps: model building, model evaluation, problem presentation, and visual feedback (20).

In this study, the optimal model selected above was used to fit the HFRS case data from 2005 to 2021, and then was used to predict the data of 2022. Then, root mean square error (RMSE) and mean absolute error (MAE) were used to evaluate the fitting and prediction performances of the model, and the smaller the values of the indicators, the better the model performance. The SARIMA model was constructed using the “tseries” and “forecast” packages in R software (version 4.2.1, R Foundation for Statistical Computing, Vienna, Austria), and the “prophet” package was used to construct the prophet model.

## 3 Results

### 3.1 General characteristics

**Figure 1:**
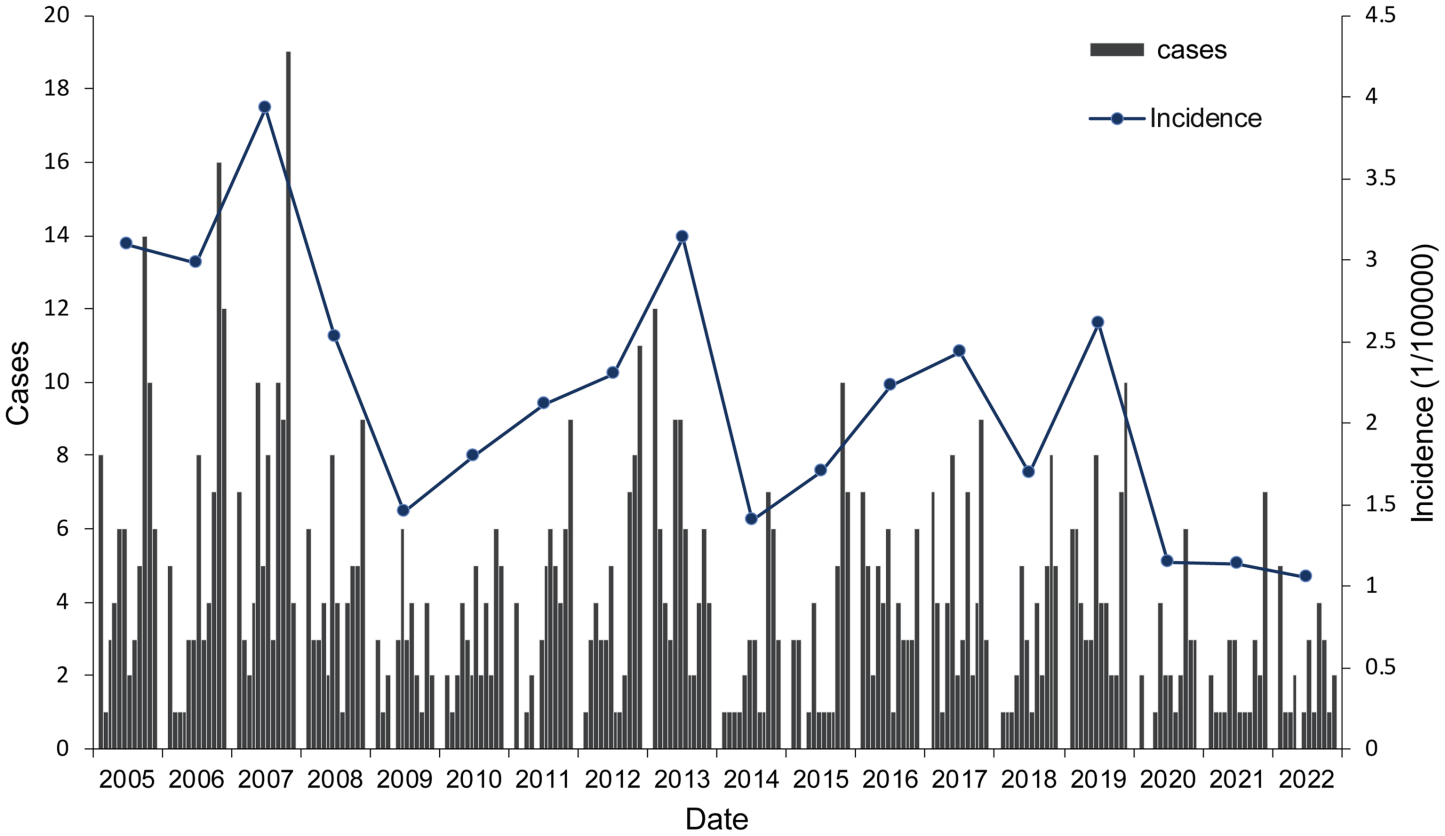
Cases and incidence rate of HFRS in Quzhou City from 2005 to 2022.

A total of 843 cases of HFRS were reported in Quzhou from 2005 to 2022, with the number of cases reported annually fluctuating in the range of 24–84. The annual average incidence was 2.15/100,000, with the lowest in 2022 (1.05/100,000) and the highest in 2007 (3.93/100,000). During the study period, the incidence decreased in a fluctuating manner, with a statistically significant difference across different years (χ^2^ = 112.791, *p* < 0.001), as shown in Figure 1. Seasonal bimodal distribution of cases, with the first peak in October–January (48.64%) and the second peak in May–July (24.79%). Among the HFRS cases, 584 were males (69.28%) and 259 were females (30.72%), with a sex ratio of 2.25:1. The incidence of HFRS in males (2.87/100,000) was higher than that in females (1.32/100,000) (χ^2^ = 114.188, *p* < 0.001). The age of onset showed a single peak distribution, ranging from 4 to 91 years old, with a median age of 51 years old. The number of cases in the age group of 50 years was the highest, accounting for 29.42% (248/843). Significant difference was observed in the incidence across different age groups, with the highest incidence in the age group of 50 years, followed by the age group of 60 years, and the overall trend showed a picture of rising first and then decreasing. The main occupation included farmers 79.95% (674/843), workers 4.86% (41/843), and students 2.61% (22/843) (Table 1).

**Table 1:** Population distribution of HFRS in Quzhou City, 2005–2022.

Variables	Cases (*n* = 843)	Proportion (%)
Sex		
Male	584	69.28
Female	259	30.72
Age group (years)		
0~	2	0.24
10~	25	2.97
20~	42	4.98
30~	114	13.52
40~	189	22.42
50~	248	29.42
60~	223	26.45
Occupation		
Farmer	674	79.95
Worker	41	4.86
Student	22	2.61
Retiree	19	2.25
Migrant laborer	13	1.54
Civil service	14	1.66
Other	60	7.12

### 3.2 Spatio-temporal characteristics

**Figure 2:**
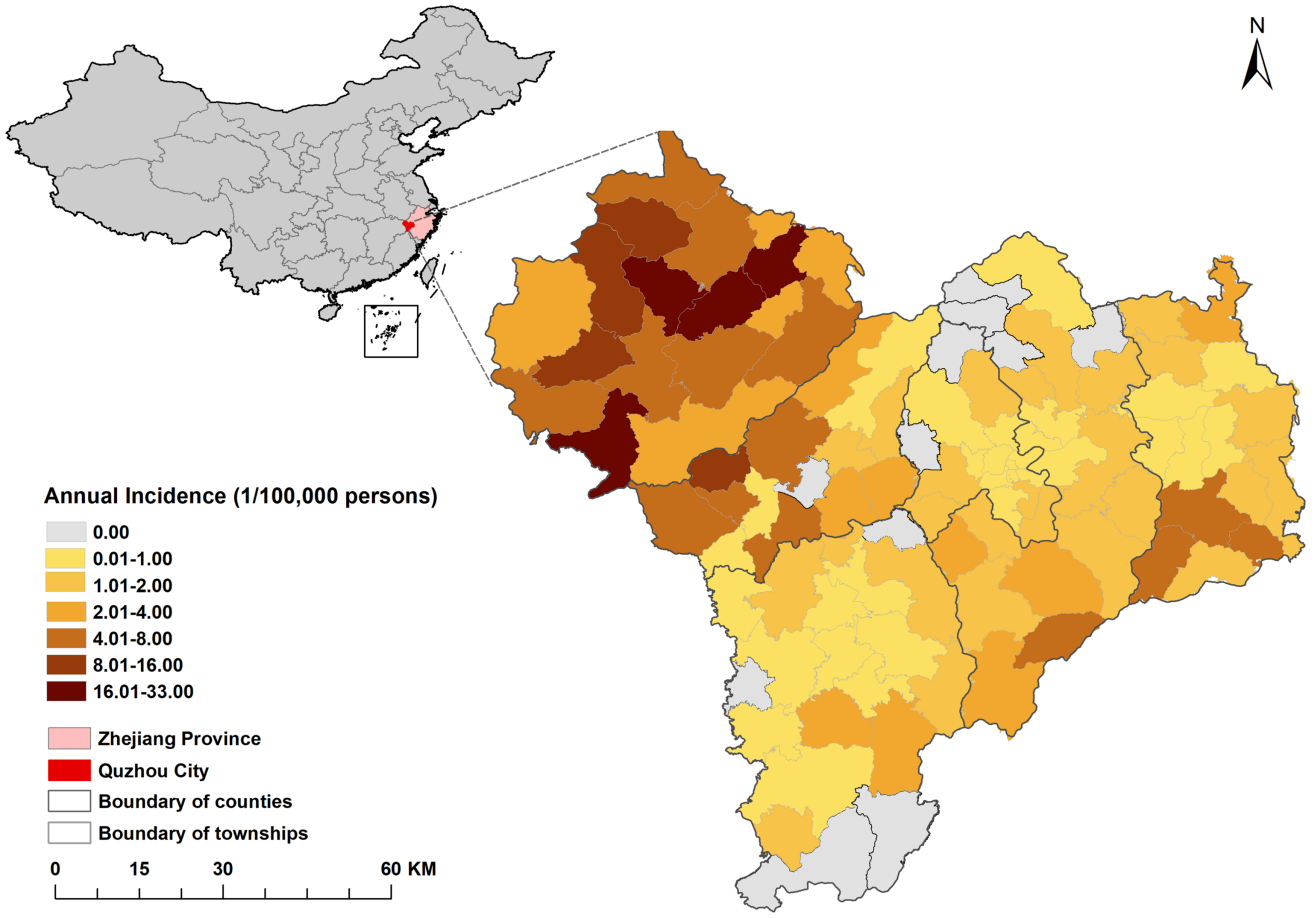
Distribution of average annual incidence rate of HFRS in each township (street) in Quzhou from 2005 to 2022.

The spatial distribution of the average annual incidence of HFRS in Quzhou from 2005 to 2022 was shown in Figure 2. The disease occurred in 6 districts and counties of the city, and the highest annual incidence was reported in Kaihua County. Among 43 streets, 39 townships, and 18 subdistricts of Quzhou, the incidence rate was highest in Yinkeng Township (32.41/100,000), followed by Cuntou Township (20.65/100,000), Zhongcun Township (17.36/100,000), and Tongcun Township (16.92/100,000). All the above townships belonged to Kaihua County. A map of incidence by year is shown in Supplementary Figure S1.

**Figure 3:**
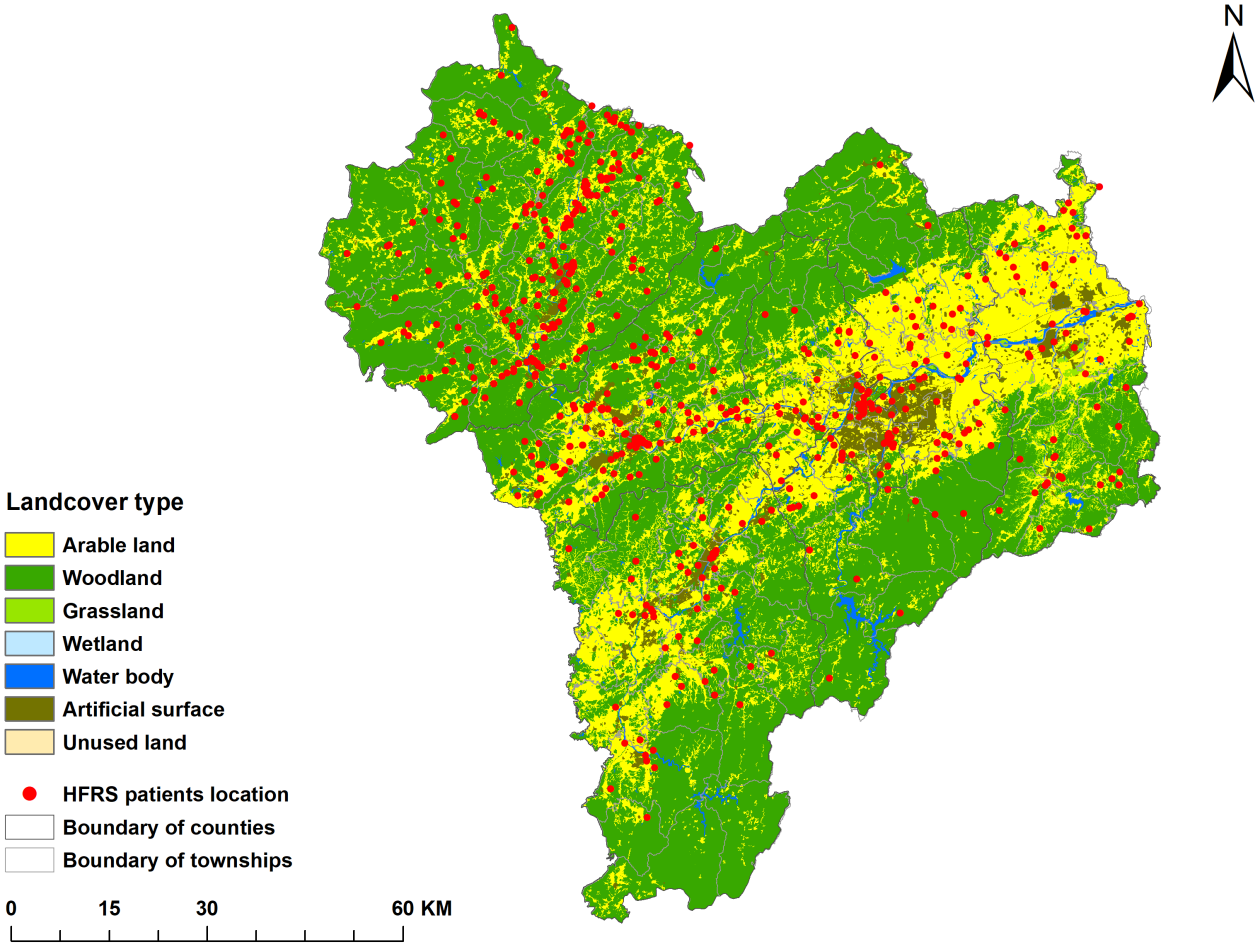
Incidence of HFRS in different land use types in Quzhou City, 2005–2022.

According to the national land resources classification system and the land use type standard, there are seven types of land use in Quzhou, namely artificial surface, woodland, water body, wetland, arable land, grassland and bare land. Combined with the cumulative incidence data from 2005 to 2022, it was found that the distribution of cases was consistent with that of cultivated land and artificial surface, as shown in Figure 3.

### 3.3 Model prediction

Using the incidence data of HFRS in Quzhou from 2005 to 2021, a time series was established in monthly basis, the time series diagram (Supplementary Figure S2) was drawn, and the component decomposition of the time series (Supplementary Figure S3) showed that the incidence of HFRS in Quzhou tended to be stable. The time series component decomposition diagram showed that the series fluctuated with seasonality, so a seasonal difference was performed on the original series. ADF test (ADF = −4.571, *p* < 0.05) showed that the sequence was stationary, that is, d = 0, D = 1. A seasonal product model with a period of 12 months was developed: SARIMA (p, 0, q) (P, 1, Q)12. The autocorrelation and partial autocorrelation analysis (Supplementary Figure S4) were carried out, q was determined to be 1, p was 1, and the values of P and Q were generally between 0 and 2. The model with the smallest AIC was selected as the optimal model by exhaustive method. It was concluded that the optimal model was SARIMA (1, 0, 1) (2, 1, 0)12, and the results showed that the model parameters were statistically significant (Table 2). The diagnostic diagram of the model (Supplementary Figure S5) showed the residual error tested by Ljung-Box test (χ^2^ = 0.241, *p* = 0.624). It was considered that the model was a white noise model with sufficient information extraction and could be used for prediction.

**Table 2:** Parameter estimation and model verification of SARIMA model.

Parameters	Estimated value	Standard error	*t*-value	*p-*value
ar1	0.855	0.077	11.052	<0.001
ma1	−0.625	0.116	−5.412	<0.001
sar1	−0.715	0.070	−10.262	<0.001
sar2	−0.389	0.071	−5.448	<0.001

SARIMA model: SARIMA (1,0,1) (2,1,0).

**Figure 4:**
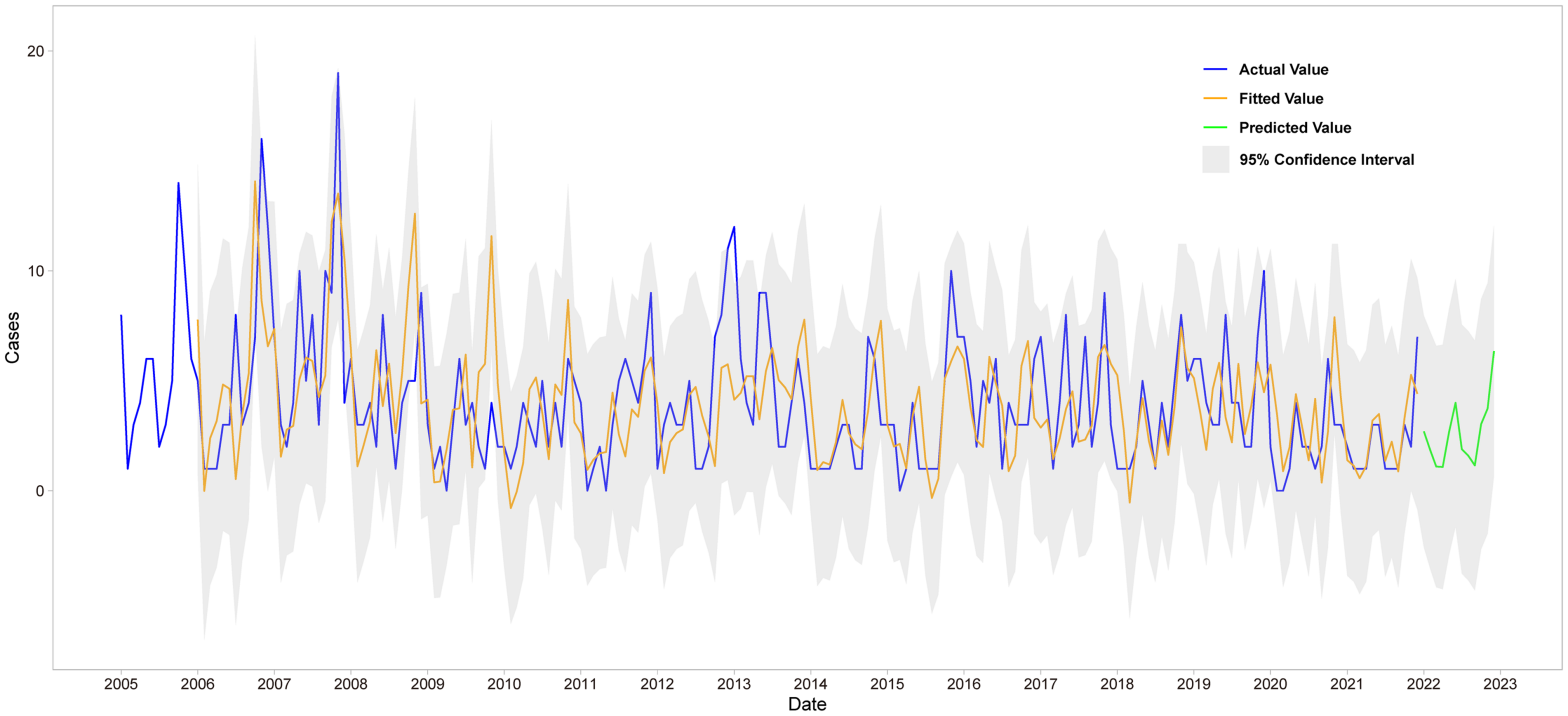
Fitting and forecast result of SARIMA.

SARIMA (1,0,1) (2,1,0)12 model was used to fit the trend of HFRS incidence in Quzhou from 2005 to 2021. The overall trend of the actual value was consistent with the fitting value, but the peak value was not fitted well. The predicted number of HFRS cases from January to December in 2022 was compared with the actual number of monthly cases, and all actual values were within the 95% CI of the predicted values (Figure 4).

**Figure 5:**
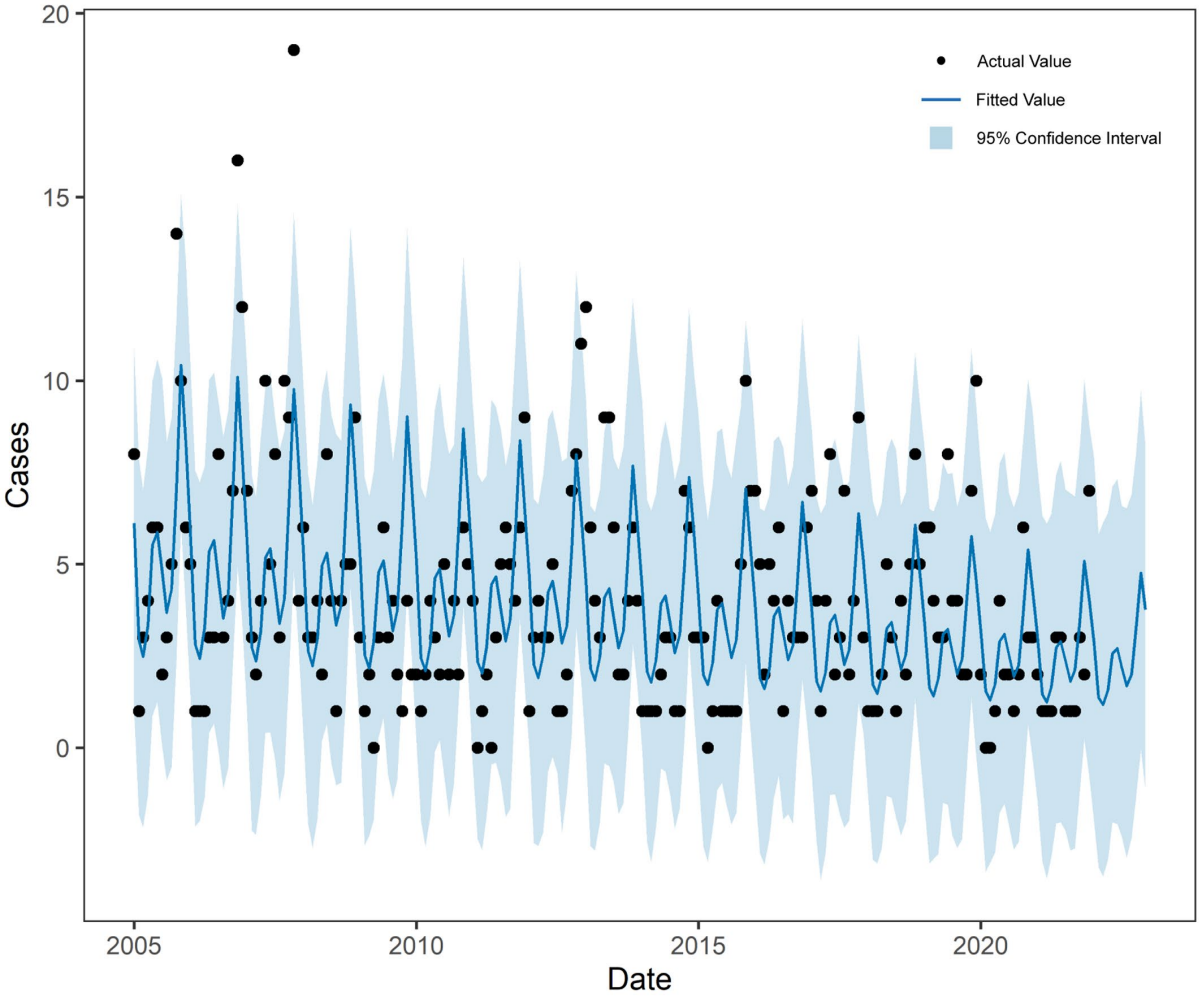
Fitting and forecast result of Prophet model.

The prophet model was constructed using the monthly incidence data of HFRS in Quzhou from 2005 to 2021 using the prophet package of R software. The interval width was set to 0.95, and other parameters were the default values. The model includes a “trend, year” time-series component (Supplementary Figure S6). Figure 5 shows that the model captures the incidence trend of HFRS well and achieves good results in the prediction of HFRS data.

The predicted values and 95% CI of SARIMA and Prophet models are shown in Table 3. The results showed that the RMSE and MAE values of the Prophet model were smaller than those of the SARIMA (1,0,1) (2,1,0)12 model in both the training set and the test set, as shown in Table 4. The Prophet model had the best performance for this time series prediction.

**Table 3:** The monthly number of HFRS cases in the Quzhou City in 2022 predicted by the SARIMA, and Prophet models.

Month	Actual values	SARIMA model		Prophet model	
		Predicted values	95% CI	Predicted values	95% CI
1	5	2.84	(−2.39, 8.07)	2.85	(−1.85, 7.31)
2	1	2.00	(−3.36, 7.37)	1.36	(−3.03, 6.20)
3	1	1.32	(−4.15, 6.78)	1.17	(−3.87, 5.96)
4	2	1.33	(−4.21, 6.87)	1.57	(−3.39, 5.95)
5	0	2.94	(−2.65, 8.53)	2.57	(−2.36, 7.24)
6	1	4.29	(−1.34, 9.91)	2.70	(−2.13, 6.98)
7	3	2.21	(−3.44, 7.86)	2.19	(−3.10, 6.71)
8	1	1.93	(−3.75, 7.60)	1.68	(−3.05, 6.22)
9	4	1.51	(−4.18, 7.20)	1.99	(−2.68, 6.48)
10	3	3.41	(−2.28, 9.11)	3.25	(−1.09, 7.72)
11	1	4.12	(−1.58, 9.82)	4.76	(0.18, 9.67)
12	2	6.73	(1.02, 12.44)	3.76	(−1.09, 8.59)

**Table 4:** Comparison of the fitting and prediction performance of the two models.

Model	Training set		Validation set	
	RMSE	MAE	RMSE	MAE
SARIMA (1,0,1) (2,1,0) (12)	2.562	1.917	2.340	1.905
Prophet model	2.393	1.847	1.754	1.388

RMSE, root mean square error; MAE, mean absolute error.

## 4 Discussion

Results from the present study showed that the incidence of HFRS in Quzhou fluctuated from 2005 to 2022, the incidence was higher in males than in females, and the occupational distribution was dominated by farmers. The main endemic area is located in the northwest of Quzhou, and cases are concentrated on cultivated land and artificial surfaces. The SARIMA and Prophet models were used for fitting and prediction, respectively, and it was found that the Prophet model performed better.

HFRS is classified as a Class B infectious disease in China according to the Law of the People’s Republic of China on the Prevention and Treatment of Infectious Diseases. In recent decades, it has been considered as one of the top 10 infectious diseases (27). Quzhou has reported HFRS cases every year. From 2005 to 2022, the average annual incidence was 2.15/100,000, which was higher than the average level of Zhejiang Province (0.91/100,000) (13). Differences in incidence due to different topography and climate (28). The city’s terrain is dominated by mountainous hills, with abundant rainfall and plenty of sunshine. It is suitable for the survival of rodents carrying HV. It has been shown that increased rodent populations can accelerate HFRS epidemics (29), and humid environments favor the breeding of mites on rodents, increasing the chances of the host carrying HV (30). From 2004 to 2015, the epidemic area of HFRS in China changed from northeast to southeast China (31). The incidence of HFRS in Quzhou was high from 2005 to 2007 and kept increasing. HV vaccination was carried out in areas such as Liaoning and Zhejiang in 2008 (32), and the incidence of the disease decreased significantly after immunization, so it is assumed that the use of the vaccine can reduce the incidence of HFRS (33). However, the epidemic began to rebound in 2010, which may be caused by changes in natural and social factors (34). In 2020–2022, the incidence of HFRS in the city was significantly lower and below average, and in 2022 it fell to the lowest value (1.05/100,000) in that study period. There are two possible reasons for the analysis: On the one hand, due to the outbreak of novel coronavirus infection at the end of 2019, the state has taken a series of non-pharmacological interventions such as quarantine, work suspension and hygiene improvement to improve people’s awareness of hygiene, develop the hygiene habit of frequent hand washing and wearing masks, and reduce garbage piling, which effectively reduced the phenomenon of mosquitoes and insects, and the rampant presence of insects and rodents (35, 36). On the other hand, the spread of COVID-19 limited the detection of other infectious diseases and reduced the behavior of seeking medical help. As a result, the incidence of HFRS was underestimated (37), which was the same as the change level of HFRS in China (38).

HFRS incidence in Quzhou City has obvious seasonality, showing a bimodal distribution with a large peak in October–January and a small peak in May–July, which is consistent with the seasonal peaks in Shandong Province and Jiangxi Province (11, 17), and a single-peak distribution in February–May in Hebei Province (39). It may be due to the different dominant species of rats in different regions, with the *Apodemus agrarius* dominated by the HTVN, which peaks in winter, while the *Rattus norvegicus* carries the SEOV with a high prevalence in the spring, which is the epidemiological season (32). Zhejiang Province is a mixed area of *Apodemus agrarius* and *Rattus norvegicus*, and the former accounts for a relatively high proportion (40), creating a dual-peak seasonal pattern that is slightly higher in winter than in spring.

The cases in all age groups were more likely to be male, and the sex difference may be caused by the difference in daily activities (41). Men have more opportunities for agricultural and outdoor labor, which is consistent with the occupational distribution characteristics of farmers (42). The over 50 age group accounted for nearly half of the total prevalence, which is related to its long history of agricultural production and lifestyle, its greater exposure to HFRS, and its greater susceptibility to the disease (43). Therefore, targeted health education on HFRS among the middle-aged and older adult population to enhance their self-protection awareness is an important measure for prevention and control.

According to the distribution map of HFRS epidemic and cumulative incidence rate in Quzhou City from 2005 to 2022, the northwestern part of Quzhou City is a high incidence area, in which Yinkeng Township, Cuntou Township, Zhongcun Township and other townships (streets) bordering Kaihua and Changshan Counties are high incidence areas. All of the above areas are mountainous and most of the population are farmers (44). Furthermore, the incidence of HFRS gradually spread from northwest to the east, which might be attributed to the accelerated urbanization process, urban expansion in the eastern part of Quzhou, and the susceptible population flocking in this area. Due to the change of land use type (45), the opportunity to contact with rodents increased, and the imperfect housing and surrounding infrastructure in the early stage increased the risk of infection (46). It is suggested that the hygiene of living environment should be improved to prevent and control rodents.

The land use distribution map showed that the risk of HFRS was concentrated in arable land and artificial surface near arable land, which was the same as the results in Hunan and Shaanxi Provinces (47, 48). Artificial land surface is the surface formed by artificial construction activities, including all kinds of residential land, industrial and mining, etc. Arable land mainly includes paddy fields, dry land, and vegetable fields. It was found that the type of land use affects the spread of HFRS by altering the environment in which rodents live, that crops are a major food source for rodents, and that farmers are more likely to come into contact with rodents during their farming practices (49). Living in areas near cropland with high rodent density increases the chance of HFRS infection (50).

In this study, based on the incidence data of HFRS in Quzhou from 2005 to 2022, the SARIMA model and Prophet model were constructed to fit and predict the incidence trend of HFRS. We find that Prophet’s optimal model is more effective as the evaluation indexes of RMSE and MAE are lower than those of SARIMA model, and the error of prediction results is smaller. SARIMA model is one of the most commonly used models in time series analysis. It has the advantages of simple structure and easy interpretation of parameters, and is widely used in the field of public health (51). However, it requires the data to be stationary series, and cannot well fit the nonlinear trend (52). HFRS morbidity information has both linear and non-linear characteristics, and SARIMA does not capture the information adequately. Taylor and Letham (26) in their analysis of hand, foot and mouth disease and Mohan et al. (53) in their study of COVID-19 found that the Prophet model had a higher predictive accuracy when using the ARIMA model versus the Prophet model to predict disease incidence. Since Prophet model has more advantages in dealing with outliers and trend migration, it can better predict the development and change of things over time (26). In practice, it is necessary to continuously adjust the model according to the new measured data to further improve the prediction ability of the model.

Our study also has limitations. First of all, the data of HFRS cases were collected by passive surveillance, which may have biases such as underreporting. Secondly, only the inherent characteristics of the time series were included in the construction of the model, and other exogenous variables, such as meteorological factors, economic factors, and rodent density, were not considered. Finally, because the study data came from Quzhou City, China, our conclusions apply only to the population in that area, and extrapolation of the results is limited. In the future, we need to explore the interactions of Prophet’s model in depth, taking into account various influencing factors, in order to obtain forecasts that are closer to reality.

## 5 Conclusion

The overall incidence of HFRS in Quzhou showed a downward trend from 2005 to 2022, but the epidemic was still serious in Cuntou town and Zhongcun Township of Kaihua County. Male middle-aged and older adult farmers should be the focus of HFRS prevention and control. In the future, we should continue to strengthen the monitoring of the epidemic dynamics and host animals of HFRS, carry out scientific rodent control and control before the onset of the high incidence season of HFRS, and promote HFRS vaccination and health education, especially for farmers. The Prophet model could well fit and predict the incidence trend of HFRS in Quzhou, and provide early warning and prediction for the occurrence of human HFRS and reliable data for the prevention and control of HFRS in Quzhou.

## Data availability statement

The original contributions presented in the study are included in the article/Supplementary material, further inquiries can be directed to BZ, bd_zhan@126.com.

## Author contributions

QG: Methodology, Visualization, Writing – original draft. SW: Data curation, Software, Writing – review & editing. QW: Writing – review & editing. GC: Writing – review & editing. CF: Writing – review & editing. BZ: Funding acquisition, Writing – review & editing.

## Footnotes

^1^ http://www.globallandcover.com/
