# Supplementary material for: Epidemiological characteristics and prediction model construction of hemorrhagic fever with renal syndrome in Quzhou City, China, 2005–2022
Source: Front Public Health. 2024 Jan 11;11:1333178. doi: 10.3389/fpubh.2023.1333178 (PMC10808376; doi:10.3389/fpubh.2023.1333178)
Supplement: Supplementary file 1 [file Data_Sheet_1.docx]

Supplementary Material

**Supplementary Figure 1.** Distribution map of annual incidence rate of HFRS in each township (street) in Quzhou from 2005 to 2022

**Supplementary Figure 2.** Time series of HFRS incidence in Quzhou City, 2005-2022

**Supplementary Figure 3.** Time Series Decomposition of HFRS Incidence in Quzhou City from 2005 to 2022

**Supplementary Figure 4.** ACF after differencing transformation (A) and PACF after differencing transformation (B)

**Supplementary Figure 5.** Residual Diagnostics Plots. Autocorrelation plot of the residual sequence (A); Partial autocorrelation plot of the residual sequence (B); Ljung-Box test plot of the residual sequence (C) and the Quantile‑Quantile Plot of residual (D).

**Supplementary Figure 6.** Decomposition of Prophet time series


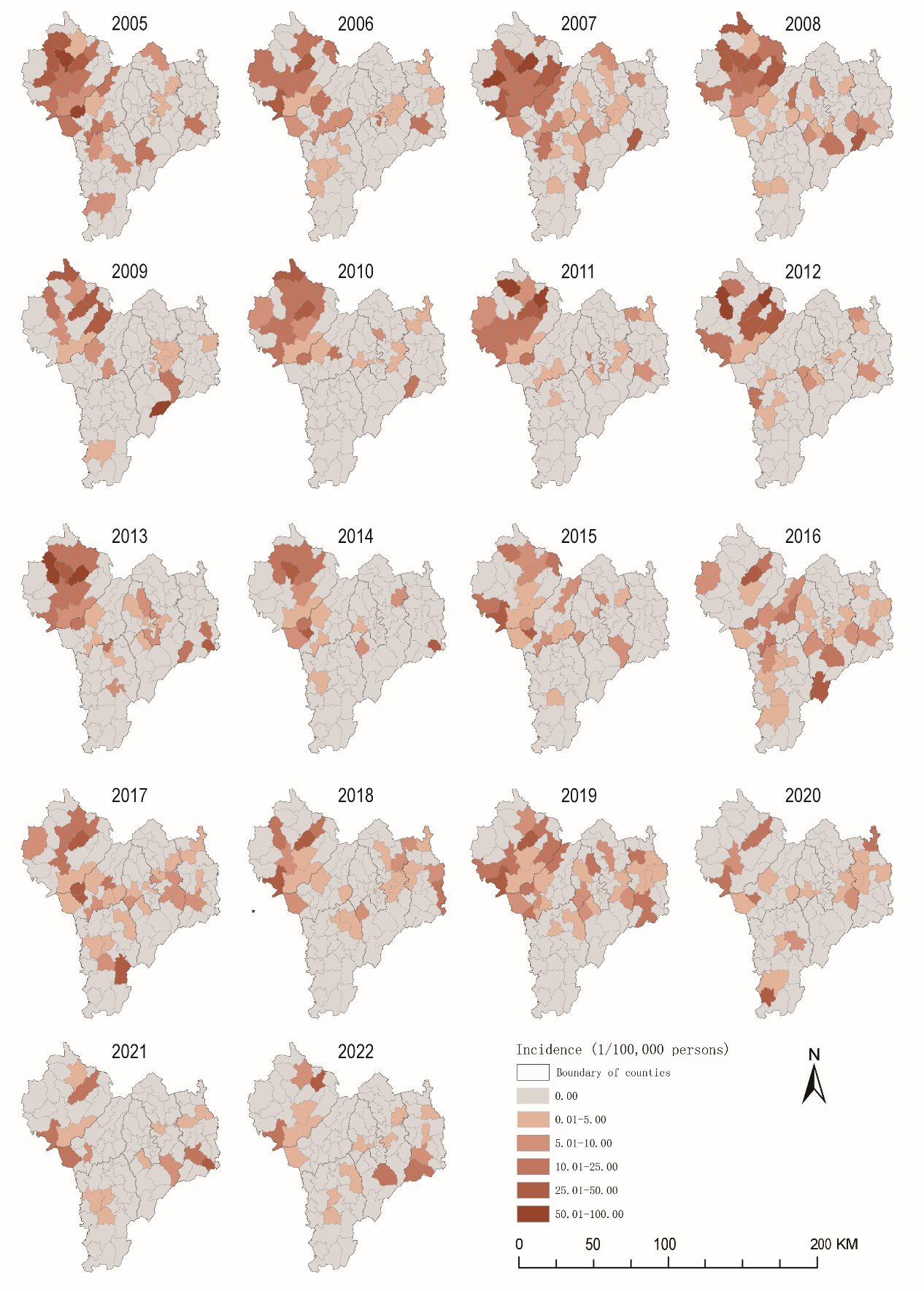


**Supplementary Figure 1.** Distribution map of annual incidence rate of HFRS in each township (street) in Quzhou from 2005 to 2022


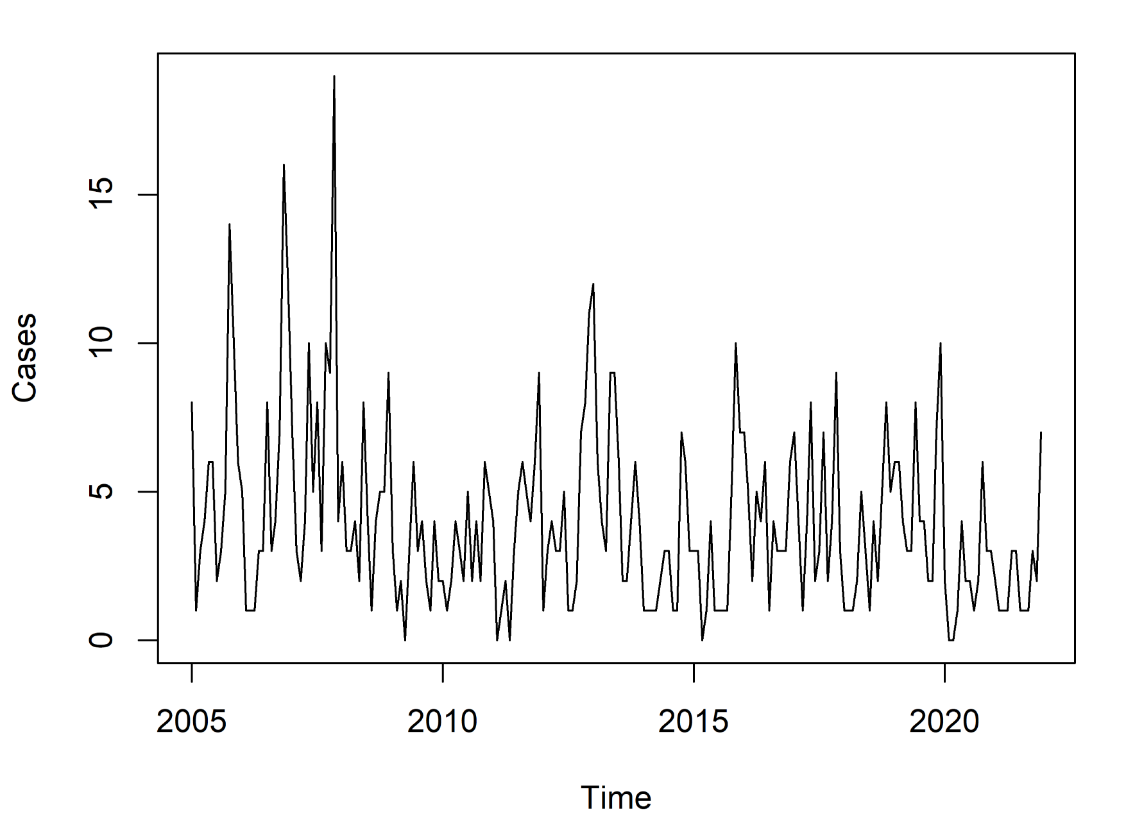


**Supplementary Figure 2.** Time series of HFRS incidence in Quzhou City, 2005-2022


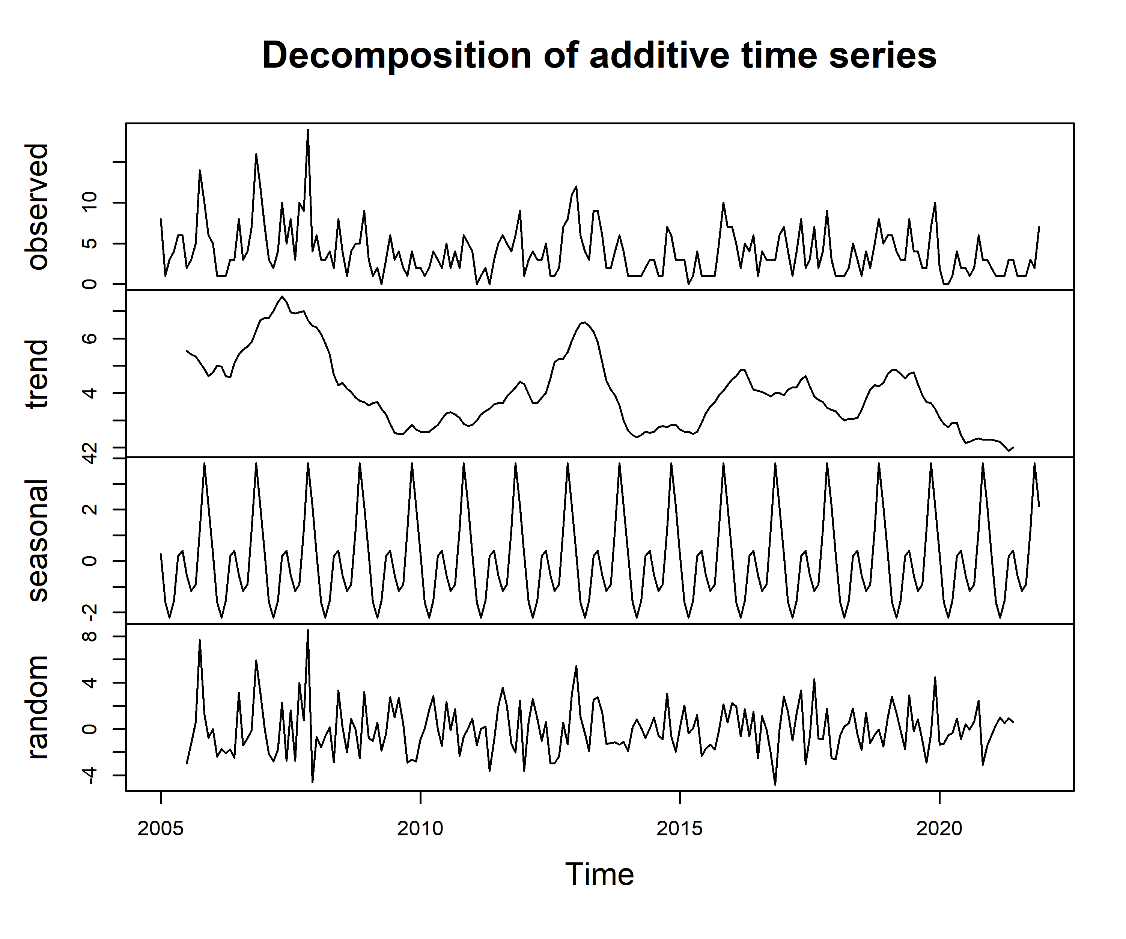
 **Supplementary Figure 3.** Time Series Decomposition of HFRS Incidence in Quzhou City from 2005 to 2022


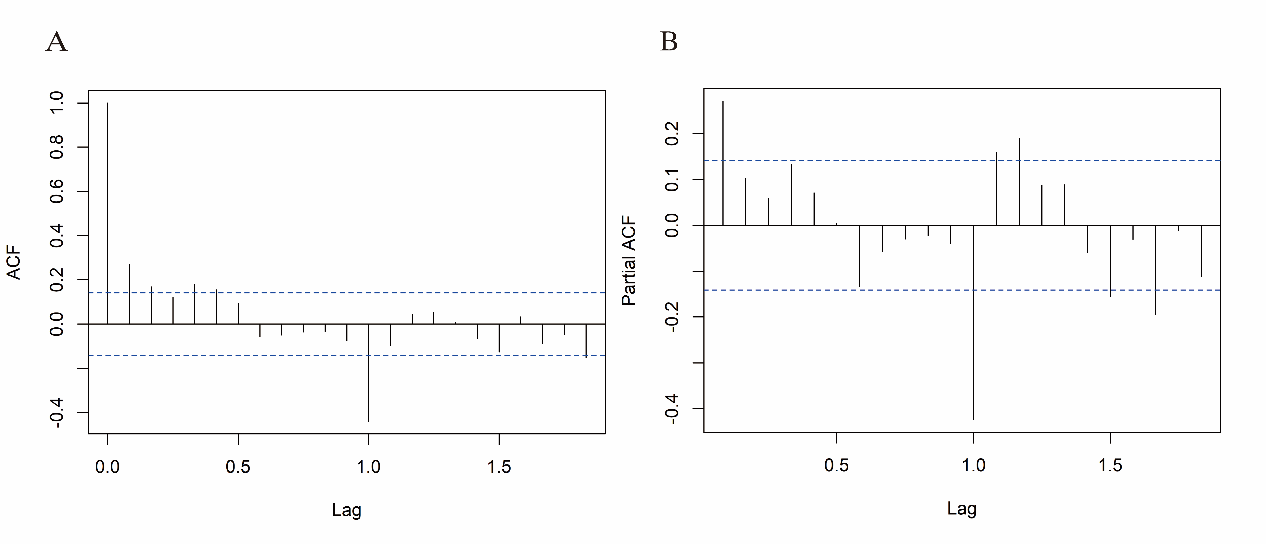


**Supplementary Figure 4.** ACF after differencing transformation (A) and PACF after differencing transformation (B)


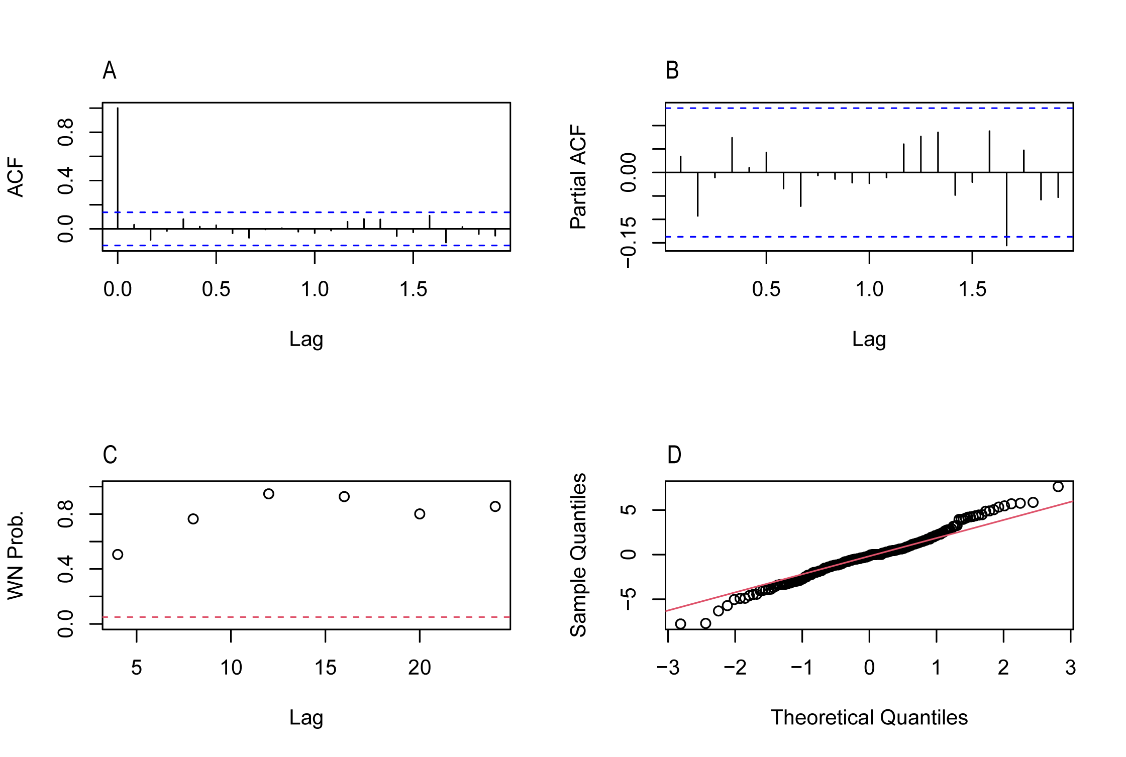


**Supplementary Figure 5.** Residual Diagnostics Plots. Autocorrelation plot of the residual sequence (A); Partial autocorrelation plot of the residual sequence (B); Ljung-Box test plot of the residual sequence (C) and the Quantile‑Quantile Plot of residual (D).


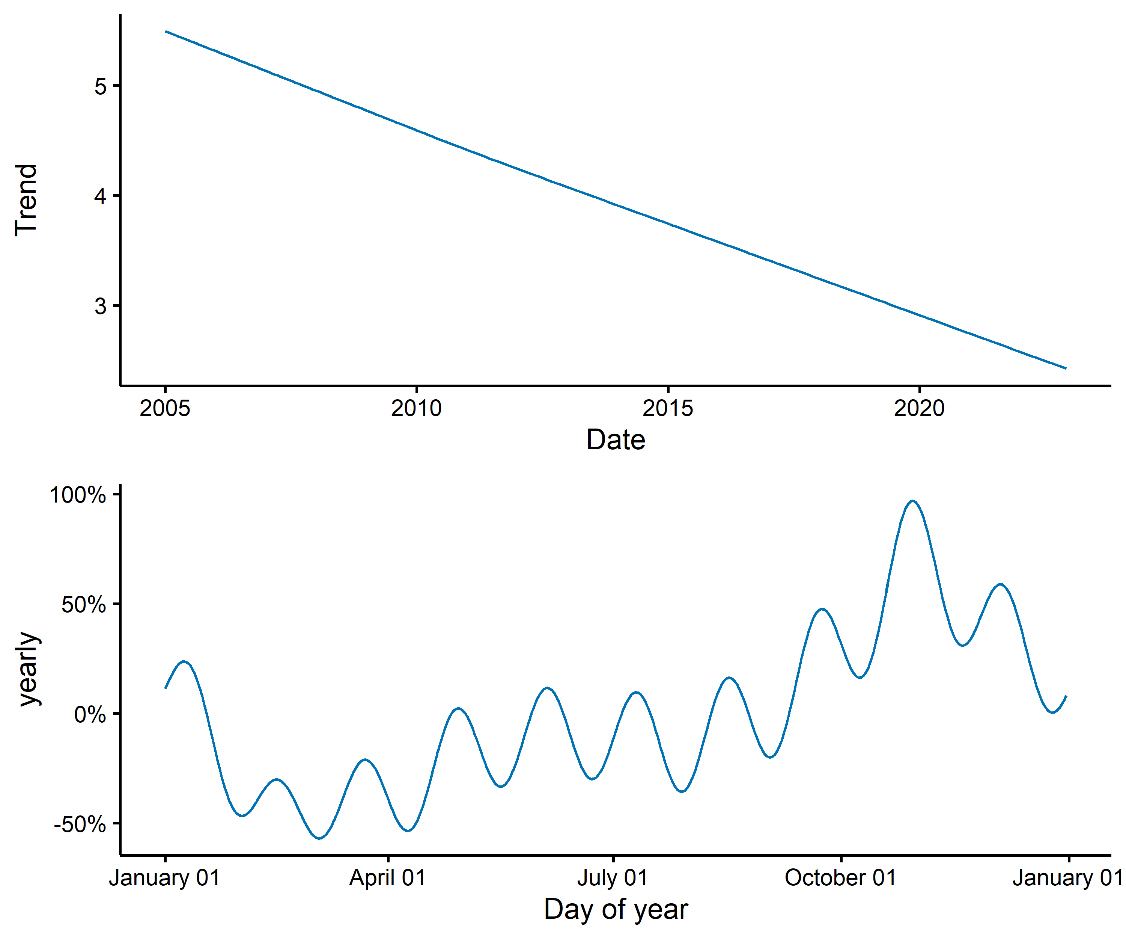
 **Supplementary Figure 6.** Decomposition of Prophet time series
